# Conformational Plasticity in the HIV-1 Fusion Peptide Facilitates Recognition by Broadly Neutralizing Antibodies

**DOI:** 10.1016/j.chom.2019.04.011

**Published:** 2019-06-12

**Authors:** Meng Yuan, Christopher A. Cottrell, Gabriel Ozorowski, Marit J. van Gils, Sonu Kumar, Nicholas C. Wu, Anita Sarkar, Jonathan L. Torres, Natalia de Val, Jeffrey Copps, John P. Moore, Rogier W. Sanders, Andrew B. Ward, Ian A. Wilson

**Affiliations:** 1 Department of Integrative Structural and Computational Biology, The Scripps Research Institute, La Jolla, CA 92037, USA; 2 Department of Medical Microbiology, Amsterdam University Medical Centers, Location AMC, University of Amsterdam, 1105 AZ Amsterdam, the Netherlands; 3 Department of Microbiology and Immunology, Weill Medical College of Cornell University, New York, NY 10021, USA; 4 IAVI Neutralizing Antibody Center, The Scripps Research Institute, La Jolla, CA 92037, USA; 5 Center for HIV/AIDS Vaccine Immunology and Immunogen Discovery, The Scripps Research Institute, La Jolla, CA 92037, USA; 6 Skaggs Institute for Chemical Biology, The Scripps Research Institute, La Jolla, CA 92037, USA

**Keywords:** HIV envelope glycoprotein, broadly neutralizing antibody, X-ray crystallography, cryoelectron microscopy, fusion peptide, ACS202, AMC011, VRC34.01

## Abstract

The fusion peptide (FP) of HIV-1 envelope glycoprotein (Env) is essential for mediating viral entry. Detection of broadly neutralizing antibodies (bnAbs) that interact with the FP has revealed it as a site of vulnerability. We delineate X-ray and cryo-electron microscopy (cryo-EM) structures of bnAb ACS202, from an HIV-infected elite neutralizer, with an FP and with a soluble Env trimer (AMC011 SOSIP.v4.2) derived from the same patient. We show that ACS202 CDRH3 forms a “β strand” interaction with the exposed hydrophobic FP and recognizes a continuous region of gp120, including a conserved N-linked glycan at N88. A cryo-EM structure of another previously identified bnAb VRC34.01 with AMC011 SOSIP.v4.2 shows that it also penetrates through glycans to target the FP. We further demonstrate that the FP can twist and present different conformations for recognition by bnAbs, which enables approach to Env from diverse angles. The variable recognition of FP by bnAbs thus provides insights for vaccine design.

## Highlights

• bnAb ACS202 penetrates the glycan shield to target the FP of HIV-1 Env gp41
• FP interacts with CDRH3 of ACS202 through a main-chain β strand interaction
• bnAbs approach Env from diverse angles to target different dispositions of FP
• FP-targeting bnAbs have varying tolerance to natural diversity in FP sequences

## Graphical Abstract

**Figure:**
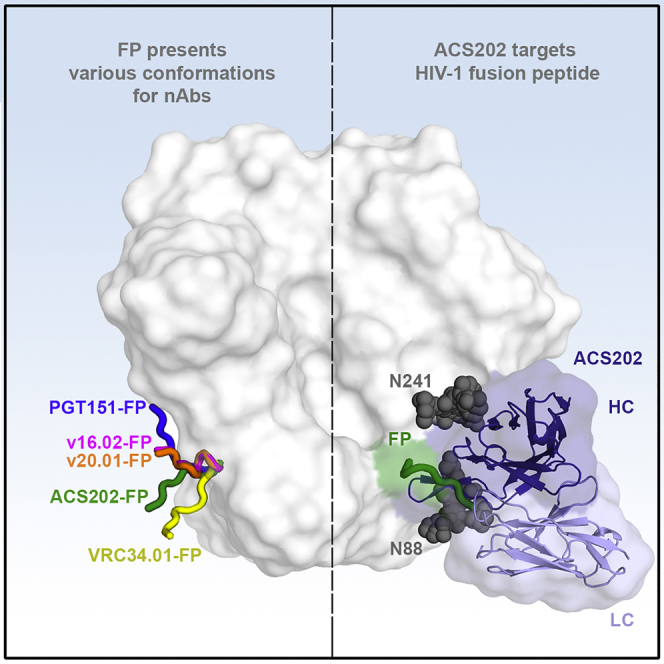

## Teaser

The HIV-1 Env fusion peptide (FP) is a site of vulnerability targeted by the immune system. Yuan et al. show that broadly neutralizing antibody ACS202 penetrates the Env glycan shield to target the FP. The diverse approach angles to the FP by different neutralizing antibodies provide insights for vaccine design.

## Introduction

The elicitation of potent broadly neutralizing antibodies (bnAbs) by vaccination is thought to be critical for protecting against HIV-1 infection. The only target for bnAbs on HIV-1 is the trimeric envelope glycoprotein (Env) spike. Numerous bnAbs to HIV-1 have been discovered, especially in the last 10 years, and have revealed an unexpectedly large number of sites of vulnerability (Chuang et al., 2019, McCoy, 2018, Sok and Burton, 2018), including the CD4-binding site, V1/V2 apex, N332/V3 base supersite, membrane-proximal external region (MPER), and, more recently, the gp120-gp41 interface.

The bnAbs targeting the gp120-gp41 interface include 8ANC195 (Scharf et al., 2014, Scheid et al., 2011), 35O22 (Huang et al., 2014), PGT151 (Blattner et al., 2014, Falkowska et al., 2014), VRC34.01 (Kong et al., 2016), and CAP248-2B (Wibmer et al., 2017). Most of these bnAbs are trimer specific and gp120-gp41 cleavage dependent. The HIV-1 Env glycoprotein is assembled as a trimer of heterodimers, with three gp120 membrane-distal subunits and three gp41 membrane-proximal and transmembrane subunits. Upon endoproteolytic cleavage of the gp160 precursor, the N-terminal region (fusion peptide, FP) of gp41 (Blumenthal et al., 2012) is liberated. The FP is hydrophobic (Figure S1A), largely disordered (Guttman et al., 2014, Kumar et al., 2019), generally but not completely conserved in sequence (Figures S1B–S1D) (Kong et al., 2016), and essential for virus entry because of its critical involvement in membrane fusion (Blumenthal et al., 2012). Extrapolation from how the FP is oriented in the pre-fusion state of the influenza hemagglutinin (HA) glycoprotein (Wilson et al., 1981) generated the hypothesis that the HIV-1 Env FP would likely be inaccessible in the gp120-gp41 interface, as a device to prevent non-specific hydrophobic interactions or premature fusion. However, bnAbs PGT151 and VRC34.01 were found to interact with the FP, as well as other components including complex glycans nearby in the gp120-gp41 interface (Lee et al., 2016) (Kong et al., 2016).

The ACS202 bnAb was isolated from an HIV-1-infected individual, AMC011, who was categorized as an “elite neutralizer” (van den Kerkhof et al., 2014, van Gils et al., 2016). AMC011 sera showed early broad HIV-1 neutralizing activity; ACS202 bnAb was isolated later in infection, exhibited 45% breadth on a panel of 87 viruses, and was shown to target the FP and N88 (van den Kerkhof et al., 2014, van Gils et al., 2016). Here, we define how bnAb ACS202 recognizes Env by determining its X-ray structure in complex with the FP and its cryo-electron microscopy (cryo-EM) structure in complex with a SOSIP.v4.2 Env trimer that was derived from the same elite neutralizer AMC011. Here, the structural studies reveal that bnAbs can take the advantage of the flexible and dynamic nature of the FP by recognizing it in multiple conformations and orientations and thereby facilitate interaction with the FP epitope by diverse antibodies, including different germlines, that can help aid in recognition and neutralization of HIV.

## Results

### Crystal Structure of ACS202 Reveals FP Recognition

A negative-stain single-particle EM reconstruction indicated that the ACS202 epitope was located in the gp120-gp41-interface (van Gils et al., 2016). Competitive binding assays with other bnAbs to the interface region, including PGT151, 35O22, and 3BC315, strongly reduced ACS202 binding to SOSIP trimers. In addition, viruses with mutations in the FP were substantially resistant to ACS202 neutralization (van Gils et al., 2016). To facilitate structural determination, we synthesized a peptide mimic of the AMC011 FP, which consisted of the first ten residues of the gp41 N terminus (residues 512–521, HXB2 numbering) and a C-terminal His_6_-tag (AVGIGAVFLGHHHHHH). A bio-layer interferometry (BLI) experiment showed that the ACS202 Fab binds the synthetic FP with a dissociation constant (*K*_D_) of 1.8 μM (Figure S1E), and binding was confirmed in an enzyme-linked immunosorbent assay (ELISA) (Figure S1F).

**Figure 1:**
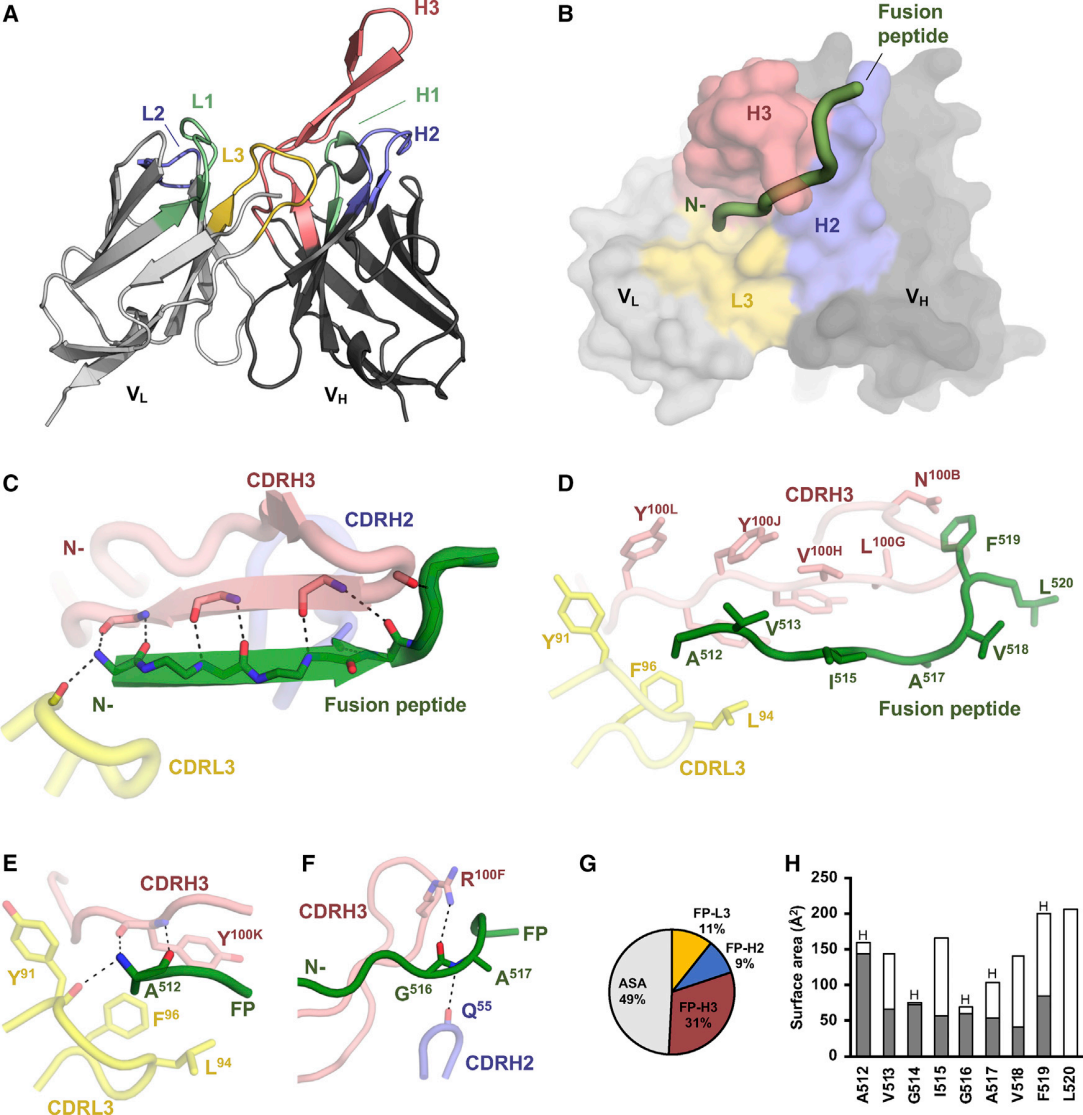
Crystal Structure of ACS202 Fab in Complex with the HIV-1 Env Fusion Peptide (A) Expanded view of the variable domains of ACS202 Fab. CDR loops are highlighted (L1/H1 in green, L2/H2 in blue, L3 in yellow, and H3 in pink). The bound FP is not shown. (B) Surface representation of the variable domains of ACS202 Fab with the FP represented by a green tube. The light- and heavy-chain variable domains are colored light and dark gray, respectively. CDR loops that are involved in FP binding are highlighted (H2 in blue, L3 in yellow, and H3 in pink). No major conformational changes were observed in the Fab on FP binding. (C) Backbone interactions between the HIV-1 FP (green) and ACS202. Hydrogen bonds between the FP and CDRL3 (yellow), H2 (blue), and H3 (pink) are shown as black dashed lines. The FP forms an antiparallel β sheet with CDRH3 of ACS202. (D) Hydrophobic interactions between FP (green) and ACS202 (CDRL3 in yellow and CDRH3 in pink). Backbones are shown as tubes, and the side chains are highlighted as sticks. (E) The ACS202 antibody intimately interacts with the N-terminal A512 of the HIV-1 FP (green). The side chain of A512 is buried in a hydrophobic pocket formed by CDRL3-Y91, L94, F96, and CDRH3-Y100^K^. Hydrogen bonds are shown as black dashed lines. (F) Stabilization of G516-A517 of the FP by ACS202. Hydrogen bonds are shown as black dashed lines. (G) Surface area of the FP. The pie chart shows that more than half of the surface area of the FP is buried by ACS202 Fab, with CDRH3 contributing to most of that interaction. Colors for each CDR loop correspond to the panels above. ASA, accessible surface area. Buried and accessible areas were calculated with PISA (Proteins, Interfaces, Structures, and Assemblies) (Krissinel and Henrick, 2007). (H) Surface area of each residue of the FP is shown in the bar chart, with buried surface area in gray and accessible area in white. Residues that form hydrogen-bond interactions with ACS202 are highlighted with “H” on top of each bar. Buried and accessible surface areas are calculated with PISA (Krissinel and Henrick, 2007). See also Figures S1 and S2 and Table S1.

We determined a crystal structure of the ACS202 Fab in complex with the synthetic FP at 2.76-Å resolution (Figure 1; Table S1). ACS202 has a 22-residue CDRH3 (Kabat numbering; Wu and Kabat, 1970; Figure 1A), which is longer than most human antibodies (Johnson and Wu, 1998) but not unusual for HIV-1 bnAbs, which often have CDRH3s that can extend from 20 to 38 residues (Sok and Burton, 2018, Yu and Guan, 2014). The hexagonal crystals contained two ACS202 Fab molecules per asymmetric unit with one Fab bound to FP (Figure S2A), whereas the other Fab was unliganded because its paratope was blocked by a symmetry mate in the crystal (Figure S2B).

The ACS202 CDRs H2, H3, and L3 form a groove that accommodates the FP in an extended conformation (Figures 1A and 1B). The electron density for the FP is well defined (Figures S2C and S2D), and all residues except 521 are visible. The FP exclusively consists of non-polar amino acids (^512^A-V-G-I-G-A-V-F-L-G^521^, Figures 1D, S2C, and S2D). The FP extends along the length of a hydrophobic groove in the ACS202 combining site (Figure 1B) and is stabilized by eight backbone-mediated hydrogen bonds, thereby forming an antiparallel β sheet with CDRH3 (Figures 1C and 1D). The N-terminal FP residue, A512, inserts into a hydrophobic pocket formed by L94 and F96 of CDRL3 together with Y100^K^ of CDRH3, in an interaction that buries more than 90% of the A512 surface area (Figure 1H). The A512 interaction is further stabilized by three main-chain hydrogen bonds to Y91 of CDRL3 and Y100^K^ of CDRH3 (Figure 1E). In CDRH3, 16 out of 22 residues are hydrophobic or aromatic (including glycines), especially on the C-terminal side of the H3 loop that is involved in binding the FP. The L100^G^-V-Y-Y-Y-Y100^L^ motif of CDRH3 forms intimate hydrophobic interactions with the FP (Figure 1D). The phenyl ring of F519 of the FP is further stabilized by a polar-π interaction (Dougherty, 2013) with the side-chain amine of CDRH3 N100^B^ (Figure 1D). The side chain of CDRH3 R100^F^ forms an additional hydrogen bond with the carbonyl oxygen of FP G516, which is further stabilized by interaction of its main-chain amide with the backbone of CDRH2 Q55 (Figure 1F). More than half of the total FP surface is buried in the interface with ACS202 and dominated by interactions with CDRH3 (Figure 1G). Each residue from A512 to F519 of the FP is buried by ACS202 ranging from 30% (V518) to 99% (G514) of the surface area, while L520 is completely exposed (Figure 1H), suggesting that ACS202 specifically recognizes only the first eight FP residues.

The FP is an essential and highly conserved functional element of the HIV-1 Env trimer (Figure S1); thus, if it is accessible, it is potentially a vulnerable site for antibodies to target. The most diverse residues within the HIV-1 FP are 515 and 518, where the hydrophobic amino acids found at these positions can vary (Figure S1D) (Crooks et al., 2004). Among available HIV-1 sequences, the most common residue at position 515 is isoleucine (51.6%), while at position 518, valine is found in 43.6% of the sequences. Sequence diversity, particularly at these two positions, may therefore limit the neutralization breadth of Abs targeting the FP region. Thus, we individually introduced the less prevalent residues into the FP of the BG505-Env pseudovirus and assessed their impact on neutralization by ACS202, PGT151, and VRC34.01. In general, ACS202 and PGT151 were more tolerant of FP diversity at positions 515 and 518 than VRC34.01 (Figure S3A). Binding assays involving the synthetic FPs confirmed this finding (Figure S3B). In particular, mutations at residue 518 substantially reduced binding and neutralization of VRC34.01, while ACS202 and PGT151 showed similar binding and neutralization. Inspection of the FP-complexes of ACS202, PGT151, and VRC34.01 indicated that the side chain of residue 518 is inserted within a hydrophobic pocket in VRC34.01 but, in contrast, is exposed in the ACS202 and PGT151 complexes (Figures S3C, S3D, and S3F). These findings imply that ACS202 and PGT151 have higher tolerance to FP diversity. We also found that V513A substitution (reflecting sequence differences found in a small percentage of HIV-1 strains) abolished FP binding of all three bnAbs (Figure S3B).

### Conserved YYYY Motif of Antibodies Accommodates the N-Terminal Region of the FP

The YYYY motif of CDRH3 of ACS202 contributes to hydrophobic interactions with the FP (Figure 1D). Despite their different binding approach angles to the Env trimer and the different FP orientations stabilized by the two bnAbs (Figure 4H), ACS202 and PGT151 adopt similar strategies for interacting with the FP. In both cases, the CDRH3 loops form antiparallel β-sheet interactions with the FP (Figures S3C–S3E), and the N-terminal A512 is buried in hydrophobic pockets formed by CDRL3 and CDRH3. For each bnAb, the CDRH3 YYYY motif makes hydrophobic stacking interactions with the N-terminal region (^512^A-V-G-I^515^) of the FP, although the motif is shifted by one residue in the respective CDRH3s (Figure S3E). These regions of ACS202 and PGT151 are encoded by a common IgHJ germline gene J6^∗^02 (Ye et al., 2013). Both bnAbs are highly conserved with their germline J gene. In both bnAbs, 18 out of 19 amino acids (95%) correspond to the germline-gene-encoded residues, including YYYY motifs (Figure S3E).

Y100^J^ makes a side-by-side interaction with FP residue V513. Within the ACS202 family of Abs (van Gils et al., 2016), the precise YYYY motif is present only in ACS202. In contrast, the corresponding motif in ACS201, 203, 204, and 205 is “YHYY” (Figure S1G); these nAbs bind less well to the FP than ACS202 (Figure S1F). A mutated version of ACS202 with Y100^J^ substituted by histidine (i.e., the YHYY motif) also bound less efficiently to AMC011 SOSIP.v4.2 (a recombinant autologous Env trimer of ACS202) and BG505 SOSIP.664 trimers, confirming the key contribution of the YYYY motif (Figures S1H and S1I). The Tyr/His polymorphism in the YYYY motif is not unique to the ACS202 family, as it is also present in PGT153-158, which are closely related to the YYYY-containing PGT151 (Falkowska et al., 2014). Despite the high flexibility and structural heterogeneity of the FP, both bnAbs ACS202 and PGT151 matured by developing the same consensus recognition features. In contrast, although VRC34.01 shares the same IgHJ germline gene J6^∗^02 with ACS202 and PGT151, it has a relatively short CDRH3 of 13 residues, which does not contain the YYYY motif but NEAV at these positions (Kong et al., 2016).

### Cryo-EM Structure Delineates the Complete Epitope of ACS202

**Figure 2:**
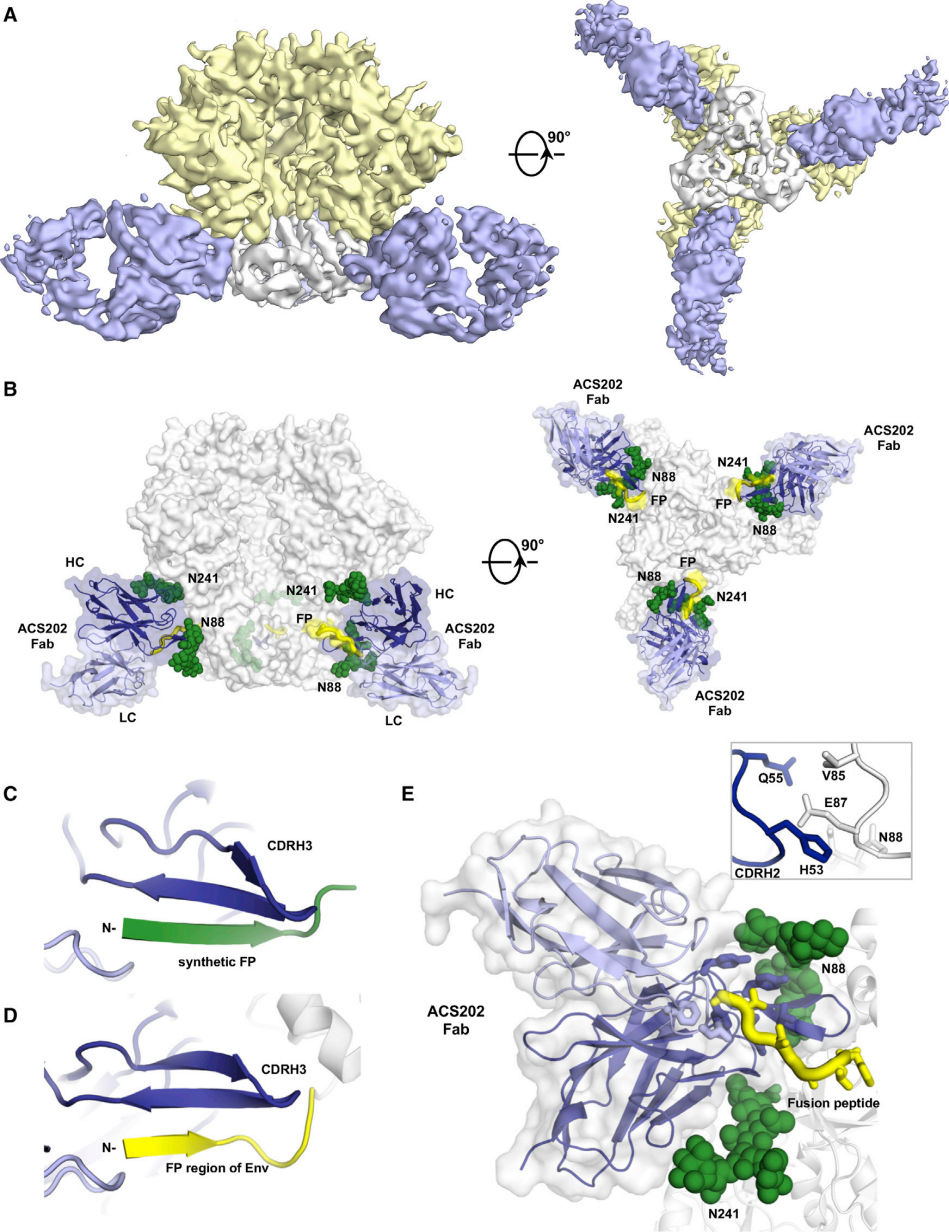
Cryo-EM Reconstruction of Env Trimer AMC011 SOSIP.v4.2 in Complex with bnAb ACS202 (A) Reconstruction of Env trimer AMC011 SOSIP.v4.2 in complex with ACS202 Fab at ∼5.2 Å resolution, segmented to highlight densities corresponding to gp120 (yellow), gp41 (white), and ACS202 Fab (blue). (B) Model of Env trimer AMC011 SOSIP.v4.2 in complex with ACS202 Fab. Glycans are shown as green spheres. The FP is shown in yellow. Variable domains of ACS202 are shown in dark (heavy chain) and light (light chain) blue. (C) Crystal structure illustrating the interaction between ACS202 (blue) and synthetic FP fragment (green). (D) Cryo-EM structure shows that the interaction between ACS202 and the FP region (yellow) of Env trimer AMC011 SOSIP.v4.2 is similar to that with the synthetic FP, as shown in (C). The root-mean-square deviation (RMSD) (Cα) between the FPs is 1.7 Å. (E) Detailed interactions of Fab ACS202 recognition of AMC011 SOSIP.v4.2 Env trimer. Glycans are shown as green spheres. Side chains of the FP (yellow) are shown in sticks. Interactions between CDRH2 of ACS202 and the Env trimer are highlighted in the top right corner. See also Figure S4 and Table S3.

The soluble, recombinant AMC011 SOSIP.v4.2 trimer is based on the consensus sequence from an early *env* gene (8 months post seroconversion) present in the ACS202 bnAb donor. ACS202 bnAb (isolated at 40 months post seroconversion) bound to this trimer with low nanomolar affinity and neutralized the consensus AMC011 virus (van Gils et al., 2016). To further investigate the structural mechanism of binding and neutralization, we determined the cryo-EM structure of ACS202 Fab in complex with AMC011 SOSIP.v4.2 to ∼5.2 Å resolution (Figures 2 and S2E).

The structure reveals a stoichiometry of Fab:gp120-gp41 protomer of 1:1 (Figures 2A and 2B). ACS202 binds perpendicular to the trimer 3-fold axis and parallel to the membrane with its heavy chain above the light chain (Figures 2A and 2B). The bnAb recognizes a very similar conformation of the FP on the Env trimer and on the synthetic FP (Figures 2C and 2D). ACS202 also interacts with other regions of the gp120-gp41 interface, including a contiguous region of gp120 (residues 85–88) and glycans at N88 and N241 (Figure 2E). The N88 glycan site is highly (>98%) conserved across different subtypes of HIV-1 Env (Figure S1D). Deletion of the N88 glycan completely abrogates ACS202 binding to the Env trimer and virus neutralization (van Gils et al., 2016).

Residue 87 within interacting residues 85–88 is highly diverse with 56% of the analyzed sequences containing glutamate, 15% glycine, 13% lysine, and 16% other amino acids (Figure S1D). The cryo-EM structure of the AMC011 SOSIP.v4.2-ACS202 complex suggests that H53^CDRH2^ and Q55^CDRH2^ make contact with E87 (Figure 2E). Compared with wild-type ACS202, the H53A, Q55A, and Q55L mutants bind less well to the AMC011 SOSIP.v4.2 and BG505 SOSIP.664 trimers (Figures S4A and S4B). Furthermore, an E87A substitution in the AMC011 SOSIP.v4.2 trimer almost completely abolishes ACS202 binding and completely abrogates neutralization of JRCSF-Env pseudovirus (van Gils et al., 2016). We assessed ACS202 binding to seven additional native-like SOSIP trimers based on sequences from subtypes A, B, and C (Figure S4D), all of which were predicted to contain the N88 glycan (Figure S4E). ACS202 was only minimally reactive with the two SOSIP trimers (B41 and ZM197M) in which residue 87 was a glycine rather than glutamate in the other five, ACS-reactive trimers. This finding is consistent with a previous neutralization study showing that all 32 ACS202-sensitive viruses contain a glutamate at position 87, while 26 of 42 non-neutralized viruses had a different residue (van Gils et al., 2016) (Figure S4F). A logistic regression analysis also showed that sensitivity to ACS202 neutralization is highly correlated with the identity of residue 87, but is not correlated for VRC34.01 and PGT151 where residue 87 is not included in their epitopes (Kong et al., 2016) (Figure S4G). Finally, we showed that a G87E substitution in the B41 SOSIP.v4 trimer partially restores ACS202 binding (Figure S4H). Taken together, the various findings confirm the critical contribution of E87 to the ACS202 epitope. The natural sequence variation seen at this position would then appear to limit the neutralization breadth of ACS202 and could serve as an escape strategy. In fact, viruses that had escaped by mutation at position 87 were indeed found in the AMC011 individual (van Gils et al., 2016).

**Figure 3:**
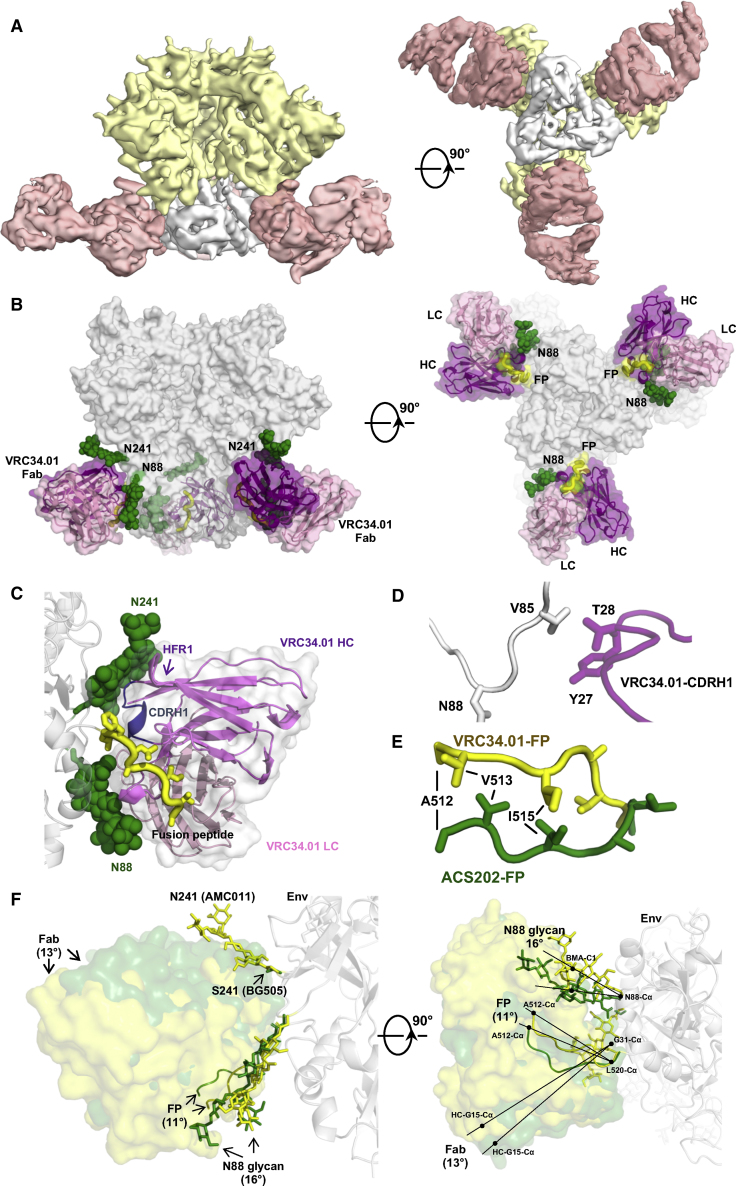
Cryo-EM Reconstruction of Env Trimer AMC011 SOSIP.v4.2 in Complex with bnAb VRC34.01 (A) Cryo-EM reconstruction of Env trimer AMC011 SOSIP.v4.2 in complex with VRC34.01 Fab at ∼4.5 Å resolution, segmented to highlight densities corresponding to gp120 (yellow), gp41 (white), and VRC34.01 Fab (pink). (B) Cryo-EM structure of the Env trimer AMC011 SOSIP.v4.2 in complex with VRC34.01 Fab. Glycan components of the epitope are shown as green spheres. The FP part of the epitope is shown in yellow. Variable domains of VRC34.01 are shown in dark (heavy chain) and light (light chain) purple. (C) Detailed interactions of VRC34.01 recognition of the FP and glycans N88 and N241. Glycans are shown as green spheres. Side chains of the FP (yellow) are shown in sticks. CDRH1 of VRC34.01 that interacts with the glycans at N241 is highlighted in blue. (D) Interactions between VRC34.01-CDRH1 (purple) with Env trimer AMC011 SOSIP.v4.2 (white). (E) Comparison between the different relative conformations of the ACS202-bound FP (green) and the VRC34.01-bound FP (yellow). gp41 molecules from the ACS202/Env complex and VRC34.01/Env complex structures were superimposed with PyMOL to show the different conformations and orientation of ACS202-bound and VRC34.01-bound FPs. (F) Comparison between the structures of VRC34.01 complexed with HIV-1 Env trimers AMC011 SOSIP.v4.2 (yellow) and BG505 SOSIP.664 (green). The epitopes on AMC011 SOSIP.v4.2 (including FP and glycans at N88 and N241), as well as the bound VRC34.01 Fab, are shown in yellow, and those of BG505 SOSIP.664 are in green, with the Env trimer in white. BG505 SOSIP.664 has serine at position 241 and thus lacks a glycan at this site. The slight angle shifts between the FPs, glycans, and Fabs of the two structures are highlighted with arrows. To measure the angle differences, Env protomers of AMC011 SOSIP.v4.2 and BG505 SOSIP.664 were aligned with PyMOL. The method to assess angle differences is shown in the right panel. See also Figures S2 and S3 and Table S3.

Residue 85 is also diverse across HIV-1 strains (Figure S1D). The valine present in the AMC011 trimer at this position is involved in contacts with ACS202 (Figure 2E), and AMC011 virus neutralization was abrogated when V85 was changed to tryptophan (van Gils et al., 2016). The VRC34.01 epitope also involves residue 85 (Figure 3D) and point substitutions at this position create VRC34.01 escape mutants (Dingens et al., 2018). The FP has been shown to elicit cross-reactive neutralizing antibodies (nAbs) in animal studies when used as an immunogen (Xu et al., 2018), and these FP-elicited antibodies were also sensitive to single-site changes at residue 85 (Dingens et al., 2018). Taken together, all known nAbs that recognize the FP, except for PGT151 (Dingens et al., 2019, Lee et al., 2016), recognize their trimer epitopes through interaction with residues 85–88. Although the glycan site at N88 is highly conserved, the nearby residues at 85 and 87 are diverse; as these residues are critical for bnAb recognition, sequence variation here may limit the breadth of anti-FP nAbs.

ACS202 binding to Env proteins is highly cleavage dependent and does not bind to the uncleaved BG505 NFL Env construct (Figure S4C). Our structures show that the first FP residue is embedded within the antibody interface, and hydrogen bonds are made from the antibody to the free amino group of the FP. These N-terminal interactions are not possible on an uncleaved trimer like NFL (Sharma et al., 2015, Yang et al., 2018), where the FP is still covalently attached to the gp120 subunit via a linker and oriented away from the gp120-gp41 interface.

### ACS202 and VRC34.01 Penetrate the HIV Glycan Shield

HIV-1 Env is heavily glycosylated, with N-linked glycans comprising roughly half the mass of the glycoprotein. Collectively referred to as the glycan shield, the numerous glycans protect sites of vulnerability as an immune evasion strategy. Here, we considered whether this feature may also shield the FP from antibody recognition, but we found that ACS202 recognizes and penetrates the glycan shield at glycans N88 and N241 and is thus able to access and target the FP (Figure 2). Previous studies have shown that VRC34.01 recognizes glycan N88 on the BG505 SOSIP.664 trimer (Kong et al., 2016). The BG505 virus lacks a glycan site at position 241 that is highly (>97%) conserved among global isolates (McCoy et al., 2016). Knocking in a glycan at BG505 residue 241 does not alter VRC34.01 neutralization sensitivity, implying that this epitope is not shielded by a glycan at this position (Dingens et al., 2018). We generated a 4.5 Å cryo-EM reconstruction of VRC34.01 with the same trimer (AMC011 SOSIP.v4.2) as in the ACS202 cryo-EM complex, which naturally contains a glycan at residue 241 (Figures 3A, 3B, and S2F). The reconstruction shows that VRC34.01 does indeed penetrate between the N241 and N88 glycans to interact with the FP (Figure 3C). The structure also demonstrates that VRC34.01 binds the FP in a very similar conformation in the AMC011 trimer to that identified in the crystal structure of VRC34.01 with the BG505 SOSIP.664 trimer (PDB: 5I8H) (Kong et al., 2016) (Figures 3F, S2G, and S2H). However, the relative disposition of the FP on the Env surface differs and, therefore, the angle of approach of the antibodies. In the AMC011 trimer structure, the FP and the N88 glycan are reoriented by ∼11° and 16°, respectively, relative to the BG505.664 structure, and the binding angle of VRC34.01 shifts by ∼13° to avoid a clash with the additional glycan present at N241 (Figure 3F). Thus, anti-FP nAbs can alter their approach angle to penetrate through the glycan shield and, thereby, target this FP site of vulnerability.

## Discussion

**Figure 4:**
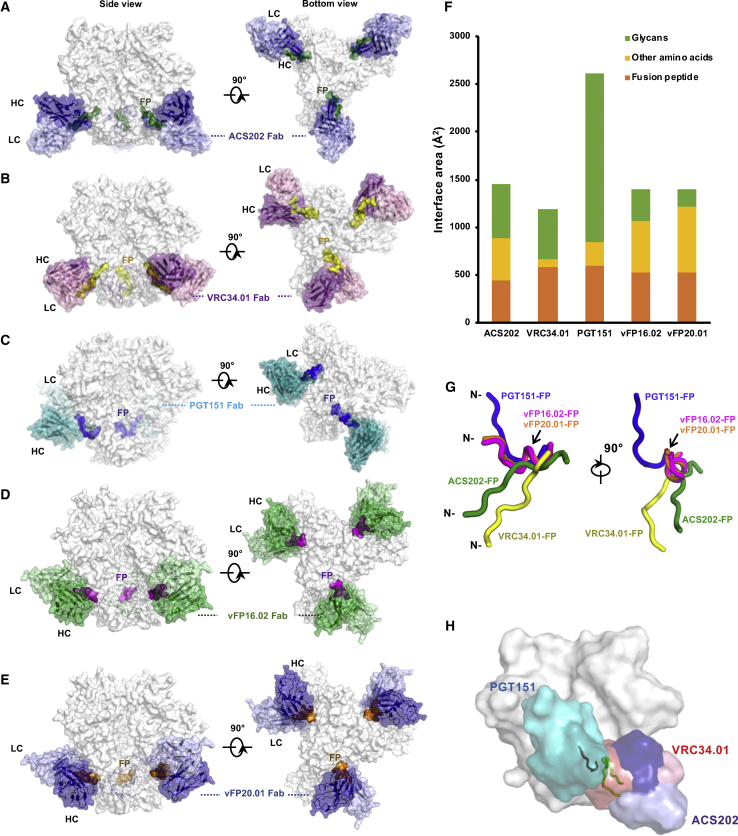
Neutralizing Antibodies Bind to HIV-1 Fusion Peptides with Different Angles of Approach (A) Cryo-EM reconstruction of Env trimer AMC011 SOSIP.v4.2 (white) in complex with ACS202 Fab (blue) shows a stoichiometry of three Fabs per trimer. The FP is shown in green. (B) Cryo-EM reconstruction of Env trimer AMC011 SOSIP.v4.2 (white) in complex with VRC34.01 Fab (purple) with a stoichiometry of three Fabs per trimer. The FP is shown in yellow. (C) Cryo-EM reconstruction of Env trimer JR-FL EnvΔCT (white) in complex with PGT151 Fab (cyan) with a stoichiometry of two Fabs per trimer (PDB: 5FUU) (Lee et al., 2016). The FP is shown in blue. (D) Cryo-EM reconstruction of Env trimer BG505 SOSIP (white) in complex with v16.02 Fab (green) with a stoichiometry of three Fabs per trimer (PDB: 6CDI) (Xu et al., 2018). The FP is shown in purple. (E) Cryo-EM reconstruction of Env trimer BG505 SOSIP (white) in complex with v20.0 Fab (blue) with a stoichiometry of three Fabs per trimer (PDB: 6CDE) (Xu et al., 2018). The FP is shown in orange. (F) Interface areas between anti-FP antibodies ACS202, VRC34.01, PGT151, vFP16.02, vFP20.01, and Env trimers. Interface area (Å^2^) with the FPs, other amino acids, and glycans are shown in orange, yellow, and green, respectively. The interface areas were calculated with PISA (Krissinel and Henrick, 2007). (G) Conformations of the FPs stabilized by ACS202 (green), VRC34.01 (yellow), PGT151 (blue), vFP16.02 (purple), and vFP20.01 (orange). Protomers of the antibody-bound Env proteins were superimposed with PyMOL, and the different conformations of the FP (A512–L520) are shown in the panel. The vFP16.02-bound and vFP20.01-bound FPs overlap with each other. (H) Comparison of the binding approaches of ACS202 (HC: dark blue, LC: light blue, bound FP: green), VRC34.01 (HC: dark red, LC: light red, bound FP: yellow), and PGT151 (HC: dark cyan, LC: light cyan, bound FP: blue) to the Env surface. See also Figure S3 and Table S2.

Our X-ray and cryo-EM structural studies reveal how ACS202 binds to the FP of the HIV-1 envelope protein. Previously, two bnAbs, PGT151 and VRC34.01, have been shown to include the FP as a component of their overall epitopes (Kong et al., 2016, Lee et al., 2016). An upward orientation of the FP is stabilized when PGT151 binds to a native Env trimer (Figures 4C and 4G, summarized in Table S2); the antibody also interacts with complex glycans at N611 and N637 on gp41 of the adjacent protomer (Lee et al., 2016). The light chain is located above (i.e., more membrane-distal) the heavy chain and is mainly responsible for the glycan interactions. The heavy-chain-light-chain axis is more perpendicular to the membrane than VRC34.01. PGT151 binds to Env in an unusual asymmetric manner, with a maximum of two Fabs per trimer, presumably because of PGT151-induced allosteric effects that occlude the third binding site (Lee et al., 2016). In contrast, the VRC34.01 binding angle is more parallel to the membrane plane and the antibody engages a downwardly oriented FP (Figures 4B and 4G). As a result, and unlike PGT151, three VRC34.01 Fab molecules can bind symmetrically to each Env trimer. In addition to the FP, N88-glycans on the same protomer are bound by VRC34.01. Notwithstanding, PGT151 and VRC34.01 both stabilize the FP in an extended conformation. Recently, the FP was used as an immunogen to elicit mouse nAbs vFP16.02 and vFP20.01 (Figures 4D and 4E) that have neutralization breadth of approximately 30% (Xu et al., 2018) and stabilize a U-shaped conformation of the FP (Figures 4G, S3G, and S3H).

ACS202 binds Env in a different mode to HIV-1 Env compared to these other bnAbs. When ACS202 binds, the FP is stabilized in an extended conformation with a slightly downward orientation that falls roughly between the PGT151 upward and the VRC34.01 downward orientations (Figures 4A and 4G). Compared to the VRC34.01-stabilized FP (Figure S3F), ACS202 recognizes the FP in an inverted orientation (rotation of ∼180° along the FP extended structure) (Figures 3E and S3C). Thus, the FP not only can adopt multiple positions and orientations but can also twist, thereby creating different conformations for recognition by nAbs. All known human anti-FP bnAbs to date also specifically recognize cleaved trimers, which is explained by the involvement of the free N-terminal residue, including the backbone, in their epitopes. PGT151 recognizes glycans, which occupy its major paratope interface, to a much greater extent than other anti-FP nAbs VRC34.01, vFP16.02, vFP20.01, and ACS202. Taken together, the flexibility of the FP and its ability to adopt different conformations and orientations (Gabrys et al., 2013, Kong et al., 2016, Lee et al., 2016, Sackett et al., 2014, Xu et al., 2018), allows nAbs from different V_H_ and V_L_ germlines (Table S2) to bind with different approach angles and to incorporate a range of other peptide and glycan components (e.g., glycans at N88, N241, N611, and N637) in their epitopes. In addition, here, we show that the neutralization breadth of anti-FP nAbs is limited by multiple factors, including the natural diversity in FP sequences in gp41 and sensitivity to mutations on the gp120 component of the epitope. These factors should be systematically considered when designing vaccines based wholly or in part on the FP.

Since the first description of the HIV-1 Env trimer crystal and cryo-EM structures in 2013 (Julien et al., 2013, Lyumkis et al., 2013), many more have been obtained, involving a range of antibodies, subtypes, and at different levels of resolution (Chuang et al., 2019, Ward and Wilson, 2017). The resulting body of information underpins our current understanding of the structure, mechanism, and biological role of Env and its recognition by bnAbs. Only some of the Env structures have experimentally observed density for the entire FP (Dingens et al., 2018, Kong et al., 2016, Kumar et al., 2019, Lee et al., 2016, Rantalainen et al., 2018, Sarkar et al., 2018, Stewart-Jones et al., 2016, Xu et al., 2018). Despite its intrinsic flexibility, it has been essential to ascertain the range of possible conformations and locations of the FP to improve FP-epitope targeting. Here, our structure of the ACS202/Env complex describes a previously unobserved FP conformation; the FP is stabilized by an interaction with ACS202 that involves a binding angle distinct from the ones previously reported. The additional knowledge of how the FP adopts multiple conformations broadens our understanding of the range of possible antibody-recognition and antibody-elicitation strategies that can be used for HIV-1 vaccine design.

## STAR★Methods

### Key Resource Table

**Table:** 

REAGENT or RESOURCE	SOURCE	IDENTIFIER
**Antibodies**		

Monoclonal anti-HIV-1 Env ACS202	van Gils et al., 2016	GenBank: KX610471.1 (HC); KX610466.1 (LC)
Monoclonal anti-HIV-1 Env ACS202(HC)-Y100jH	This study	N/A
Monoclonal anti-HIV-1 Env ACS202(HC)-H53A	This study	N/A
Monoclonal anti-HIV-1 Env ACS202(HC)-Q55A	This study	N/A
Monoclonal anti-HIV-1 Env ACS202(HC)-Q55L	This study	N/A
Monoclonal anti-HIV-1 Env ACS201	van Gils et al., 2016	GenBank: KX610470.1 (HC); KX610465.1 (LC)
Monoclonal anti-HIV-1 Env ACS203	van Gils et al., 2016	GenBank: KX610472.1 (HC); KX610467.1 (LC)
Monoclonal anti-HIV-1 Env ACS205	van Gils et al., 2016	GenBank: KX610474.1 (HC); KX610469.1 (LC)
Monoclonal anti-HIV-1 Env VRC34.01	Kong et al., 2016	GenBank: KU711822.1 (HC); KU711823.1 (LC)
Monoclonal anti-HIV-1 Env PGT151	Falkowska et al., 2014	RRID: AB_2725801
Monoclonal anti-HIV-1 Env PGT124	Sok et al., 2013	RRID: AB_2725801
Monoclonal anti-HIV-1 Env VRC01	Wu et al., 2010	RRID: AB_2491019
Monoclonal anti-HIV-1 Env PGT145	Walker et al., 2011	RRID: AB_2491054
Monoclonal anti-HIV-1 Env 2G12	Kunert et al., 1998	RRID: AB_2491068
HRP-labeled goat-anti-human IgG	Jackson ImmunoResearch	Cat#109-035-097; RRID: AB_2337585

**Bacterial and Virus Strains**		

BG505 pseudovirus	Sanders et al., 2015	Genbank: DQ208458
BG505 pseudovirus (I515M)	This study	N/A
BG505 pseudovirus (V518M)	This study	N/A
BG505 pseudovirus (V518F)	This study	N/A

**Chemicals, Peptides, and Recombinant Proteins**		

AMC011 SOSIP.v4.2 envelope trimer	van Gils et al., 2016	N/A
BG505 SOSIP.664 envelope trimer	Sanders et al., 2013	N/A
BG505 SOSIP.664 NFL envelope trimer	van Gils et al., 2016	N/A
AMC008 SOSIP envelope trimer	de Taeye et al., 2015	N/A
AMC009 SOSIP envelope trimer	This study	N/A
B41 SOSIP envelope trimer	Pugach et al. 2015	N/A
B41 SOSIP envelope trimer (G87E)	This study	N/A
ZM197M SOSIP envelope trimer	Julien et al., 2015	N/A
DU422 SOSIP envelope trimer	Julien et al., 2015	N/A
Synthetic fusion peptide (AVGIGAVFLGHHHHHH)	Innopep	N/A
Synthetic fusion peptide (AIGIGAVFLGHHHHHH)	Innopep	N/A
Synthetic fusion peptide (AAGIGAVFLGHHHHHH)	Innopep	N/A
Synthetic fusion peptide (AVGLGAVFLGHHHHHH)	Innopep	N/A
Synthetic fusion peptide (AVGMGAVFLGHHHHHH)	Innopep	N/A
Synthetic fusion peptide (AVGIGAMFLGHHHHHH)	Innopep	N/A
Synthetic fusion peptide (AVGIGALFLGHHHHHH)	Innopep	N/A
Synthetic fusion peptide (AVGIGAFFLGHHHHHH)	Innopep	N/A
Synthetic fusion peptide (AVGIGAVLLGHHHHHH)	Innopep	N/A
3,3′,5,5′-tetramethylbenzidine	Sigma-Aldrich	Cat#860336
BsmBI	New England Biolabs	Cat#R0580L
DpnI	New England Biolabs	Cat#R0176L
T4 DNA Ligase	New England Biolabs	Cat#M0202L
Sodium chloride (NaCl)	Sigma-Aldrich	Cat#S9888
Tris Base	Sigma-Aldrich	Cat#11814273001
Bovine Serum Albumin (BSA)	Sigma-Aldrich	Cat#A9418
Tween 20	Fisher Scientific	Cat#BP337-500
Chemicals for protein crystalization	Hampton Research	N/A
Phosphate-buffered saline (PBS)	Thermo Fisher Scientific	Cat#2898933514040133
DMEM medium	Thermo Fisher Scientific	Cat#2898933511995065
Fetal calf serum	Life Technologies	Cat#10270106
Penicillin	Sigma-Aldrich	Cat#P3032
Streptomycin	VWR International B.V.	Cat#0382-EU-100G
DEAE-Dextran	Sigma-Aldrich	Cat#D9885
Reporter lysis buffer	Promega	Cat#E3971
H_2_O_2_	Brunschwig	Cat#CP26.1
Sodium acetate	VWR International B.V,	Cat#1.06268.1000
Citric acid	Brunschwig	Cat#5110.1
OPTI-MEM	Thermo Fisher Scientific	Cat#2898933531985070

**Critical Commercial Assays**		

KOD Hot Start DNA Polymerase	EMD Millipore	Cat#71086-3
QIAprep Spin Miniprep Kit	QIAGEN	Cat#27106
Luciferase Assay kit	Promega	Cat#E1500
NucleoBond Xtra Maxi	Clontech Laboratories	Cat#740414.100

**Deposited Data**		

Crystal structure of ACS202-FP	PDB	PDB: 6NCP
AMC011 SOSIP.v4.2-ACS202 cryo-EM map	EMDB	EMDB: EMD-0433
AMC011 SOSIP.v4.2-ACS202 coordinates	PDB	PDB: 6NC2
AMC011 SOSIP.v4.2-VRC34.01 cryo-EM map	EMDB	EMDB: EMD-0434
AMC011 SOSIP.v4.2-VRC34.01 coordinates	PDB	PDB: 6NC3

**Experimental Models: Cell Lines**		

Human: FreeStyle HEK293F cells	Thermo Fisher Scientific	Cat#R79007
Human: TZM-bl	NIH AIDS Reagent Program	Cat#8129

**Oligonucleotides**		

ACS202HC-H53A-F 5′-GTTATAGGAGGTGGTGCTGGACAGCATCAGTCT-3′	Integrated DNA Technologies	N/A
ACS202HC-H53A-R 5′-AGACTGATGCTGTCCAGCACCACCTCCTATAAC-3′	Integrated DNA Technologies	N/A
ACS202HC-Q55A-F 5′-GGAGGTGGTCATGGAGCGCATCAGTCTTATTCC-3′	Integrated DNA Technologies	N/A
ACS202HC-Q55A-R 5′-GGAATAAGACTGATGCGCTCCATGACCACCTCC-3′	Integrated DNA Technologies	N/A
ACS202HC-Q55L-F 5′-GGAGGTGGTCATGGACTGCATCAGTCTTATTCC-3′	Integrated DNA Technologies	N/A
ACS202HC-Q55L-R 5′-GGAATAAGACTGATGCAGTCCATGACCACCTCC-3′	Integrated DNA Technologies	N/A
ACS202HC-Y100jH-F 5′-GGACGGCTGGTCTATCATTATTATGGAATGGAC-3′	Integrated DNA Technologies	N/A
ACS202HC-Y100jH-R 5′-GTCCATTCCATAATAATGATAGACCAGCCGTCC-3′	Integrated DNA Technologies	N/A

**Recombinant DNA**		

pPPI4 expression vector	John Moore Laboratory	N/A
pHL-sec expression vector	Aricescu et al., 2006	Addgene Cat#99845

**Software and Algorithms**		

PyMOL	Schrödinger	RRID: SCR_000305
UCSF Chimera	Pettersen et al., 2004	RRID: SCR_004097
xia2	Winter, 2010	RRID: SCR_015746
PHASER	McCoy et al., 2007	RRID: SCR_014219
SWISS-MODEL	Arnold et al., 2006	RRID: SCR_014224
Phenix	Adams et al., 2010	RRID: SCR_014224
Coot	Emsley and Cowtan, 2004	RRID: SCR_014222
Relion	Kimanius et al., 2016	RRID: SCR_016274
Rosetta	Frenz et al., 2019	RRID: SCR_015701
Leginon software suite	Suloway et al., 2005	RRID: SCR_016731
MotionCor2	Zheng et al., 2017	RRID: SCR_016499
GCTF	Zhang, 2016	RRID: SCR_016500
DoG Picker	Voss et al., 2009	RRID: SCR_016655
CryoSPARC	Punjani et al., 2017	RRID: SCR_016501
Modeler	Eswar et al., 2006	RRID: SCR_008395
CARP	Lütteke et al., 2005	RRID: SCR_009021
Privateer	Agirre et al., 2015	www.ccp4.ac.uk/html/privateer.html
EMRinger	Barad et al., 2015	fraserlab.com/2015/02/18/EMringer/
MolProbity	Chen et al., 2010	RRID:SCR_014226
PISA	Krissinel and Henrick, 2007	RRID: SCR_015749
Graphpad Prism	GraphPad	RRID: SCR_002798

**Other**		

HiLoad 16/600 Superdex 200-pg column	GE Healthcare	Cat#28989335
2G12 5-ml column made in-house using NHS-activated HP resin and 2G12 IgG	This study	N/A
PGT145 5-ml column made in-house using using NHS-activated HP resin and PGT145 IgG	This study	N/A
Protein A affinity column	GE Healthcare	Cat#2898933517040301
Kappa select affinity column	GE Healthcare	Cat#17545812
CF-2/2–4 C cryoEM grids	Electron Microscopy Sciences	Cat#CF-224C-100
n-dodecyl-β-D-maltopyranoside (DDM)	Anatrace	Cat#D310 25 GM
Ni-NTA biosensors for bio-layer interferometry assays	ForteBio	Cat#18-5102
Protein G biosensors for bio-layer interferometry assays	ForteBio	Cat#18-5083

### Contact for Reagent and Resource Sharing

Further information and requests for resources and reagents should be directed to and will be fulfilled by the Lead Contact, Ian A. Wilson (wilson@scripps.edu).

### Experimental Model and Subject Details

#### Cell Lines

HEK293F cells (Life Technologies) were utilized for the production of HIV-1 Env proteins, Fabs, and IgGs. TZM-bl cells (NIH AIDS reagent program) were used for neutralization experiments. The sex of both cell lines are female.

### Method Details

#### Protein Expression and Purification

BG505 SOSIP.664 and AMC011 SOSIP.v4.2 trimers were expressed in 293F cells (Life Technologies) and affinity purified using 2G12 or PGT145 IgG cross-linked sepharose columns. Briefly, cells were co-transfected with SOSIP and furin plasmids using a ratio of 4:1, and 293Fectin (Invitrogen) as the transfection reagent. After seven days, the cells were harvested and the supernatant passed over 2G12 or PGT145 affinity columns. Trimers were eluted with 3 M MgCl_2_ pH 7.4, and further purified by size exclusion using a HiLoad 16/600 Superdex 200 pg column (GE Healthcare) in 20 mM Tris pH 7.4, 150 mM NaCl (TBS).

Antibody IgGs and Fabs were transiently transfected in FreeStyle HEK 293F cells (Invitrogen) and expressed with a ratio of 2:1 (HC:LC). After 5-6 days, cells were harvested and supernatant collected. IgGs were purified using Protein A columns (GE Healthcare). Fabs were purified using Kappa select column (GE Healthcare) followed by cation exchange chromatography (GE Healthcare), and further purified by size exclusion chromatography.

#### Purification of AMC011 SOSIP.v4.2-Fab Complexes

Env trimers were incubated with a 10x molar excess of Fab overnight at room temperature. The following morning, each complex was purified using a HiLoad 16/600 Superdex 200pg size exclusion column (GE Healthcare) with Tris-buffered saline (50 mM Tris pH 7.4, 150 mM NaCl) as the running buffer, and the peak corresponding to trimer-Fab complex was pooled and concentrated to ∼5 mg/mL.

#### Enzyme-Linked Immunosorbent Assay for Protein or Peptide Binding

ELISAs were performed as described previously (Derking et al., 2015, Sanders et al., 2013, van Gils et al., 2016). Briefly, Microlon 96-wells plates (Greiner Bio-One, Alphen aan den Rijn, The Netherlands) were coated overnight with mAb D7324 (Alto BioReagents, Dublin, Éire) at 10 μg/ml in 0.1 M NaHCO_3_, pH 8.6 (50 μl/well) for the SOSIP trimer binding assay. Ni-NTA plates (EN) were used for the FP peptide (AVGIGAVFLGHHHHHH) binding assay. Both D7324-coated plates and Ni-NTA plates were blocked using TBS (150 mM NaCl, 20 mM Tris) plus 2% skimmed milk. After washing, purified D7324-tagged SOSIP proteins (2.5 μg/ml) or FP peptides (2.5 μg/ml) were added in TBS/2% milk for 2 h. Unbound protein or peptide was washed away by two wash steps with TBS, followed by serially diluted mAbs in TBS/2% skimmed milk added for 2 h and followed by three washes with TBS. Horseradish peroxidase labeled goat-anti-human immunoglobulin G (IgG) (Jackson Immunoresearch, Suffolk, England) was diluted 3000-fold into TBS/2% skimmed milk and added for 2 h, followed by five washes with TBS/0.05% Tween20. Colorimetric detection was performed using a solution containing 1% 3,3’,5,5’-tetramethylbenzidine (Sigma-Aldrich, Zwijndrecht, The Netherlands), 0,01% H_2_O_2_, 100 mM sodium acetate and 100 mM citric acid. Color development was stopped using 0.8 M H_2_SO_4_ after 5 min, and absorption was measured at 450 nm. ELISAs were conducted with duplicate measurements.

#### TZM-bl Based Neutralization Assays

Neutralization experiments were carried out as described previously (Derking et al., 2015, Sanders et al., 2013, van Gils et al., 2016). In summary, one day prior to infection, TZM-bl cells (NIH AIDS reagent program) were plated on a 96-well plate in DMEM containing 10% FCS, 1× MEM nonessential amino acids, penicillin and streptomycin (both at 100 U/ml), and incubated at 37°C in an atmosphere containing 5% CO_2_. TZM-bl cells were not authenticated in the laboratory, but were periodically tested for mycoplasma contamination. Virus (500 pg) was incubated for 60 min at room temperature with threefold serial dilutions of monoclonal antibodies. This mixture was added to the cells and 40 μg/ml DEAE, in a total volume of 200 μl. Two days later, the medium was removed and lysed in Reporter Lysis Buffer (Promega, Madison, WI). Luciferase activity was measured using a Luciferase Assay kit (Promega, Madison, WI) and a Glomax Luminometer according to the manufacturer's instructions (Turner BioSystems, Sunnyvale, CA). Uninfected cells were used to correct for background luciferase activity. Nonlinear regression curves were determined and IC_50_ values were calculated using a sigmoid function in Graphpad Prism v5.01. Neutralization experiments were conducted with triplicate measurements.

#### Bio-layer Interferometry Binding Analysis

Binding measurements between antibodies and antigens were carried out on an Octet Red instrument (ForteBio). For the determination of the binding between FPs and Fabs, C-terminally His_6_-tagged FPs were associated to Ni-NTA sensors (ForteBio) in kinetic buffer (1× TBS pH 7.4 containing 0.002% Tween20 and 0.01% BSA) for 300 seconds at 28°C. Data were analyzed using the ForteBio analysis software version 7.1 (ForteBio) and the kinetic parameters were calculated using a global fit 1:1 model. For determination of the binding between IgGs and Env trimers, IgGs were associated to Protein G sensors (ForteBio) in kinetic buffer (1× TBS pH 7.4 containing 0.002% Tween20 and 0.01% BSA) for 300 seconds at 28°C. All bio-layer interferometry experiments were conducted a minimum of three times.

#### Crystallization and Structure Determination

A mixture of 6.5 mg/ml of purified ACS202 Fab and 5× (molar ratio) C-terminally His-tagged FP (AVGIGAVFLGHHHHHH) was screened for crystallization using the 384 conditions of the JCSG Core Suite (Qiagen) at both 277 and 293 K using our custom-designed robotic CrystalMation system (Rigaku) at TSRI by the vapor diffusion method in sitting drops containing 0.1 μl of protein and 0.1 μl of reservoir solution. Optimized crystals were then grown in 1.6 M ammonium sulfate and 0.1 M bicine pH 8.7. Crystals were flash cooled in liquid nitrogen with 25% (v/v) glycerol as a cryoprotectant. Diffraction data were collected at cryogenic temperature (100 K) at beamline 23-ID-B of the Argonne Photon Source (APS) with a beam wavelength of 1.033 Å, and processed with xia2 (Winter, 2010). Structures were solved by molecular replacement using PHASER with an homology model for Fab ACS202 generated from PDB ID: 4ZYK (Gilman et al., 2015) with SWISS-MODEL (Arnold et al., 2006). Iterative model building and refinement were carried out in COOT (Emsley and Cowtan, 2004) and PHENIX (Adams et al., 2010), respectively.

#### Cryo-EM Data Collection and Processing

n-dodecyl β-D-maltoside (DDM; Anatrace) was added to a final concentration of 0.06 mM to both purified trimer-Fab complexes. A 3-μL aliquot of the complex was applied to a C-Flat grid (CF-2/2–4C, Electron Microscopy Sciences, Protochips), which had been plasma cleaned for 10 s using a mixture of Ar/O2 (Gatan Solarus 950 Plasma system), and samples were vitrified using either a manual plunger (ACS202 Fab complex) or an FEI Vitrobot system (VRC34 Fab complex).

The samples were imaged using an FEI Titan Krios electron microscope (Thermo Fisher) operating at 300 kV and a Gatan K2 Summit direct electron director operating in counting mode. Automated data collection was performed using the Leginon software suite (Suloway et al., 2005). Each micrograph movie was collected at a magnification of 29,000x, which resulted in a pixel size of 1.03 Å in the specimen plane. Data collection information and statistics for each sample are summarized in Table S3. Micrograph movie frames were aligned and dose-weighted using MotionCor2 (Zheng et al., 2017), and CTF models were calculated using GCTF (Zhang, 2016).

Single particles were selected using DoG Picker (Voss et al., 2009) from the whole-frame aligned and summed micrographs, and particles extracted using Relion 2.1 (Kimanius et al., 2016) using a box size of 288 pixels (ACS202 Fab complex) or 352 pixels (VRC34 Fab complex). 2D and 3D classifications were performed using a combination of Relion 2.1 (Kimanius et al., 2016) and CryoSPARC (Punjani et al., 2017). The most abundant particles for each complex were “dimers of trimers”, caused by light chain interactions between 2-fold symmetry-related Fabs. This interaction resulted in D3 symmetry of the entire complex, in which the bases of two trimers face one another (but do not interact). Final reconstructions were performed in Relion 2.1 with D3 symmetry imposed, and after post-processing, the final resolution estimates (FSC 0.143) are ∼5.2 Å for AMC011 v4.2 SOSIP in complex with ACS202 Fab and ∼4.5 Å for AMC011 v4.2 SOSIP in complex with VRC34 Fab. Additional data processing statistics are summarized in Table S3.

Atomic models were built and refined into the high-resolution reconstructions by creating homology models using Modeller (Eswar et al., 2006), followed by iterative cycles of manual building in COOT (Emsley and Cowtan, 2004), real space refinement in Phenix 1.13 (Adams et al., 2010) and real space refinement using Rosetta Relax (DiMaio et al., 2009). Glycans were refined in Rosetta (Frenz et al., 2019) and validated by CARP (Lütteke et al., 2005) and Privateer (Agirre et al., 2015), and the overall structures were evaluated using EMRinger (Barad et al., 2015) and MolProbity (Chen et al., 2010). Final model statistics are summarized in Table S3.

### Quantification and Statistical Analysis

Statistical models inherent to Relion 2.1 (Kimanius et al., 2016) and CryoSPARC (Punjani et al., 2017) were employed in image analysis to derive 2D classes and 3D models. All binding and neutralization assays were conducted with at least duplicate measurements.

### Data and Software Availability

All data generated or analyzed during this study are included in this published article (and its Supplemental Information). Atomic coordinates and structure factors of the reported crystal structure have been deposited in the Protein Data Bank (PDB: 6NCP). Cryo-EM reconstructions have been deposited in the Electron Microscopy Data Bank (EMDB: EMD-0433, EMD-0434), and in the Protein Data Bank (PDB: 6NC2, 6NC3).

## Supplemental Information

Document S1. Figures S1–S4 and Tables S1–S3

Document S2. Article plus Supplemental Information
