# Supplementary material for: Conformational Plasticity in the HIV-1 Fusion Peptide Facilitates Recognition by Broadly Neutralizing Antibodies
Source: Cell Host Microbe. 2019 Jun 12;25(6):873–883.e5. doi: 10.1016/j.chom.2019.04.011 (PMC6579543; doi:10.1016/j.chom.2019.04.011)
Supplement: Document S1. Figures S1–S4 and Tables S1–S3 [file mmc1.pdf]

Cell Host & Microbe, Volume *25*

Supplemental Information

Conformational Plasticity in the HIV-1 Fusion Peptide Facilitates Recognition by Broadly Neutralizing Antibodies

Meng Yuan, Christopher A. Cottrell, Gabriel Ozorowski, Marit J. van Gils, Sonu Kumar, Nicholas C. Wu, Anita Sarkar, Jonathan L. Torres, Natalia de Val, Jeffrey Copps, John P. Moore, Rogier W. Sanders, Andrew B. Ward, and Ian A. Wilson

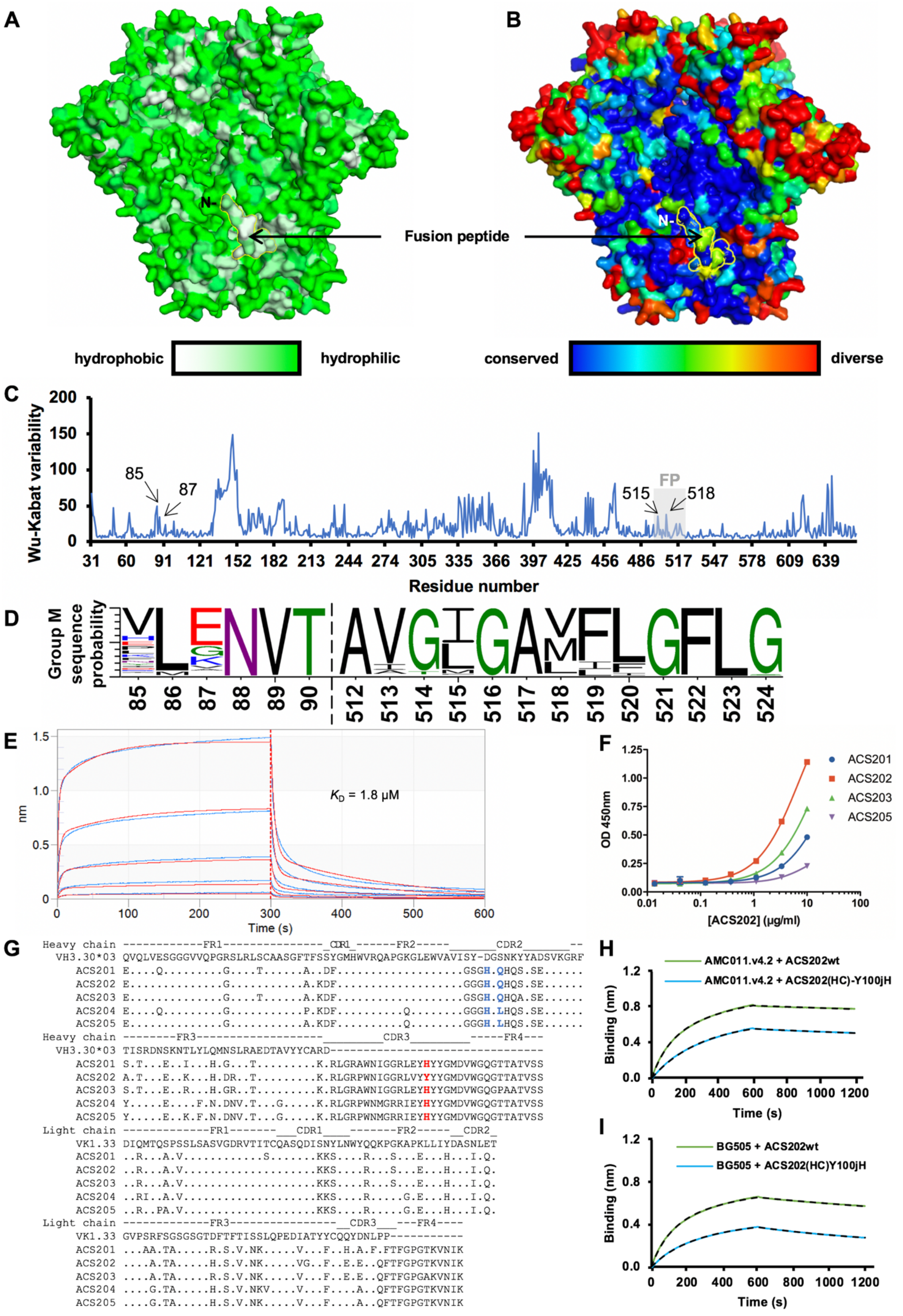

A
B
N-
Fusion peptide
hydrophobic
hydrophilic
conserved
diverse
C
Wu-Kabat variability
200
150
100
50
0
85
87
515
518
FP
31 60 91 121 152 182 213 244 274 305 335 366 397 425 456 486 517 547 578 609 639
Residue number
D
Group M sequence probability
85 86 87 88 89 90 512 513 514 515 516 517 518 519 520 521 522 523 524
E
nm
1.5
1.0
0.5
0
K_D = 1.8 μM
0 100 200 300 400 500 600
Time (s)
F
OD 450nm
1.25 1.00 0.75 0.50 0.25 0.00
ACS201
ACS202
ACS203
ACS205
0.01 0.1 1 10 100
[ACS202] (μg/ml)
G
Heavy chain ------------FR1---------------_CDR1_------FR2-----_______CDR2_______--
VH3.30*03 QVQLVESGGGVVQPGRSLRLSCAASGFTFSSYGMHWVRQAPGKGLEWVAVISY-DGSNKYYADSVKGRF
ACS201 E....Q.........G....T......A..DF...................GSGH.QHQS.SE......
ACS202 E..............G...........A.KDF...................GGGH.QHQS.SE......
ACS203 E..............G....T......A.KDF...................GSGH.QHQA.SE......
ACS204 E....Q.........G...........P.KDF..........Q........GGGH.LHQS.SE......
ACS205 E..............G...........A.KDF..........Q........GGGH.LHQS.SE......
Heavy chain --------FR3------------------_________CDR3_________-----FR4---
VH3.30*03 TISRDNSKNTLYLQMNSLRAEDTAVYYCARD--------------------------------
ACS201 S.T...E..I...H.G...T.........K.RLGRAWNIGGRLEYHYYGMDVWGQGTTATVSS
ACS202 A.T...E..K...H.DR..T.........K.RLGRPWNIGGRLVYYYYGMDVWGQGTTATVSS
ACS203 S.T...E..R...H.GR..T.........K.RLGRAWNIGGRLEYHYYGMDVWGQGPAATVSS
ACS204 Y.....E....F.N.DNV.T...G.....K.RLGRPWNMGRRIEYHYYGMDVWGQGTTATVSS
ACS205 Y.....E..K.F.N.DNV.T...G.....K.RLGRPWNMGRRIEYHYYGMDVWGQGTTATVSS
Light chain -------------FR1-------___CDR1____-------FR2-----__CDR2_
VK1.33 DIQMTQSPSSLSASVGDRVTITCQASQDISNYLNWYQQKPGKAPKLLIYDASNLET
ACS201 ..R...A.V...........S.......KKS....R...S...E...H...I.Q.
ACS202 ..R...A.V...................KKS....R...S...E...H...I.Q.
ACS203 ..R...A.V...................KKS....R...S...E...H...I.Q.
ACS204 ..RI..A.V...................KKS....R.....G.E...H...I.Q.
ACS205 ..R..PA.V...................KKS....R.....G.E...H...I.Q.
Light chain -------------FR3----------------__CDR3___---FR4----
VK1.33 GVPSRFSGSGSGTDFTFTISSLQPEDIATYYCQQYDNLPP-----------
ACS201 ...AA.TA.....R.S.V.NK.....V...F..H.A.F.FTFGPGTKVNIK
ACS202 ....A.TA.....H.S.V.NK.....VG..F..E.E..QFTFGPGTKVNIK
ACS203 ....A.TA.....R.S.V.NK.....V...F..E.E..QFTFGPGAKVNIK
ACS204 ....G.TA.....H.S.V.NKV....V...F....A..QFTFGPGTKVNIK
ACS205 ....G.TA.....H.S.V.NK.....V...F....A..QFTFGPGTKVNIK
H
AMC011.v4.2 + ACS202wt
AMC011.v4.2 + ACS202(HC)-Y100jH
Binding (nm)
1.2 0.8 0.4 0.0
0 200 400 600 800 1000 1200
Time (s)
I
BG505 + ACS202wt
BG505 + ACS202(HC)Y100jH
Binding (nm)
1.2 0.8 0.4 0.0
0 200 400 600 800 1000 1200
Time (s)

Figure S1. Characteristics of HIV-1 Env proteins and bnAb ACS202. Related to Figure 1.

(A) Hydrophobicity map of HIV-1 Env protein AMC011 SOSIP.v4.2 (hydrophobic: white; hydrophilic: green).

(B-D) Sequence conservation map of the Env proteins across 6223 group-M strains from the HIV database (www.hiv.lanl.gov/content/index). The Wu-Kabat variability (Wu and Kabat, 1970) values were mapped onto the HIV-1 trimer structure and color-coded in panel B and shown in trace mode in panel C. The FP region is highlighted in a gray box, with the diverse residues that interact with ACS202 pointed out with arrows. Details of the diversity is shown in panel D with sequence logo generated by WebLogo (Crooks et al., 2004).

(E) Binding kinetics of ACS202 against the C-terminal His_6-tagged FP (^{512}AVGIGAVFLG^{521}) were measured by bio-layer interferometry (BLI). Y-axis represents the response. Blue lines represent the response curve and red lines represent the best fit model (1:1 binding model, see Methods). Binding kinetics were measured with 70 μM of FP and different concentrations of ACS202 Fab (3,000 nM, 1000 nM, 333 nM, 111 nM, 37 nM, and 0 nM).

(F) Binding of ACS201, ACS202, ACS203, and ACS205 to the C-terminal His_6-tagged FP (^{512}AVGIGAVFLG^{521}) was measured by ELISA.

(G) Alignment of the IgH and IgLκ amino-acid sequences of ACS201-ACS205 with the most closely related germline and V gene regions indicated above. Residues 53 and 55 of the heavy chain are highlighted in blue, and CDRH3 residue 100^J in red. The CDR definitions are according to Kabat numbering (Wu and Kabat, 1970).

(H-I) Binding of wild-type ACS202 and CDRH3-Y100^JH to Env trimers (H) AMC011 SOSIP.v4.2 and (I) BG505SOSIP.664 were measured by bio-layer interferometry (BLI). The y-axis represents the response. Green and cyan lines represent the response curves and black dashed lines represent the best fit models (1:2 binding model, see STAR Methods).

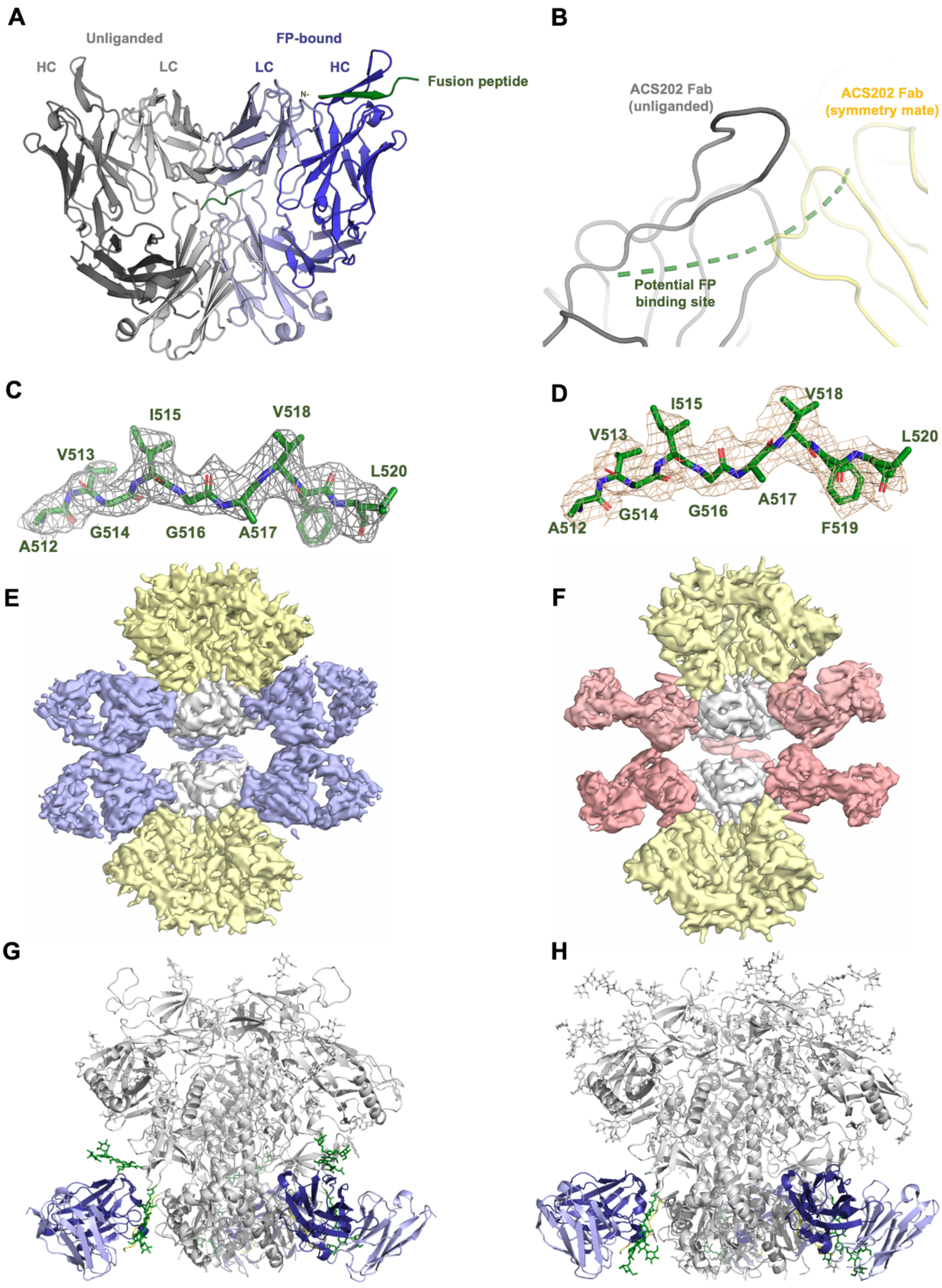
A
Unliganded
FP-bound
HC
LC
LC
HC
N-
Fusion peptide
B
ACS202 Fab
(unliganded)
ACS202 Fab
(symmetry mate)
Potential FP
binding site
C
I515
V518
V513
L520
A512
G514
G516
A517
D
I515
V518
V513
L520
A512
G514
G516
A517
F519
E
F
G
H

Figure S2. Structural details of anti-FP bnAbs and their epitopes. Related to Figures 1 and 3.

(A) Two ACS202 Fab molecules are present in the asymmetric unit of the crystal structure: an unliganded Fab molecule (gray) and an Fab (blue) complexed with the FP (green).

(B) The crystallographic symmetry mate (yellow) blocks the potential FP binding site (shown as dashed green cartoon) of the unliganded ACS202 Fab (gray). The position of the FP binding site is generated by superimposing the FP-bound Fab molecule onto the unliganded Fab molecule with PyMOL.

(C) Electron density map of the FP. The 2Fo-Fc electron density map is represented in a gray mesh contoured at 0.6σ.

(D) An Fo-Fc unbiased omit electron density map of the FP is represented in a brown mesh contoured at 1.2σ.

(E) Reconstruction of Env trimer AMC011 SOSIP.v4.2 in complex with ACS202 Fab at ~5.2 Å resolution with D3 symmetry, segmented to highlight densities corresponding to gp120 (yellow), gp41 (white), and ACS202 Fab (blue).

(F) Reconstruction of Env trimer AMC011 SOSIP.v4.2 in complex with VRC34.01 Fab at ~4.5 Å resolution with D3 symmetry, segmented to highlight densities corresponding to gp120 (yellow), gp41 (white), and VRC34.01 Fab (pink).

(G) Cryo-EM structure of VRC34.01 complexed with HIV-1 Env AMC011 SOSIP.v4.2 reconstructed at 4.5 Å. The Env trimer is shown in gray, with the epitopes highlighted (the FP in yellow, glycans in green). Heavy and light chains of VRC34.01 are shown in dark and light blue, respectively.

(H) Crystal structure VRC34.01 complexed with HIV-1 Env trimer BG505 SOSIP.664 at 4.3 Å (PDB ID: 5I8H) (Kong et al., 2016). The Env trimer is shown in gray, with the epitopes highlighted (the FP in yellow, glycans in green). Heavy and light chains of VRC34.01 are shown in dark and light blue, respectively.

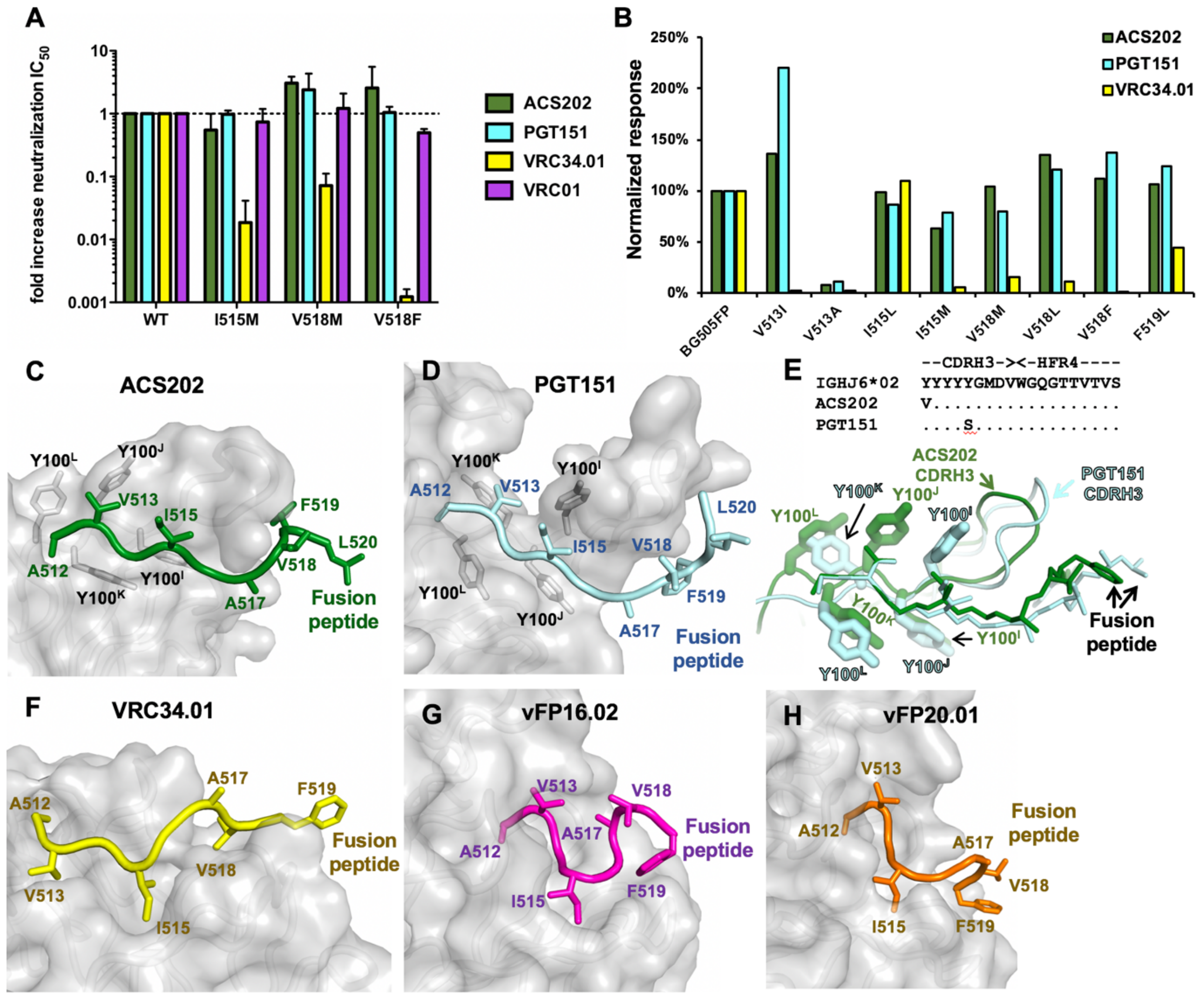

Figure S3. Neutralizing antibodies recognize the FP in different ways. Related to Figures 3E and 4.

(A) Neutralization of bnAbs ACS202, PGT151 and VRC34.01 against FP-mutated BG505 pseudoviruses. VRC01 (bnAb to gp120 CD4 binding site) was used as a control. Data represent the mean ± standard error of triplicate measurements.

(B) Binding of ACS202, PGT151 and VRC34.01 against C-terminal His_6-tagged FPs (including the BG505-derived FP ^{512}AVGIGAVFLG^{521} and other FPs with varied residues) were measured by bio-layer interferometry (BLI). The sequences of the FPs were derived from natural HIV-1 strains. Y-axis represents the response normalized to binding response of each antibody against BG505-FP.

(C) Crystal structure of ACS202 Fab (gray) in complex with FP (green).

(D) Complex structure of PGT151 Fab (gray) in complex with FP (cyan). This structure is derived from a cryo-EM structure of PGT151 Fab in complex of a native HIV-1 Env trimer JR-FL EnvΔCT (PDB ID: 5FUU) (Lee et al., 2016).

(E) Structural comparison between ACS202 Fab (green) and PGT151 Fab (cyan). Fusion peptides (represented by thin sticks) were superimposed. CDRH3 loops are highlighted with arrows. Side chains of the "YYYY" motifs in ACS202 and PGT151 are shown as thick sticks, labeled in green and cyan, respectively. Sequence alignment with the common putative germline sequence in CDRH3 and FR4 [IGHJ6*2, IMGT database (Ye et al., 2013)] is shown at the top of the panel, with identical residues represented by dots. Only 1 out of 19 residues is somatically hypermutated in each bnAb, where the first Tyr is mutated to Val in ACS202 and fifth Tyr to Ser in PGT151.

(F) Crystal structure of VRC34.01 Fab (gray) in complex with FP (yellow) (PDB ID: 5I8E) (Kong et al., 2016).

(G) Crystal structure of vFP16.02 Fab (gray, a FP-elicited mouse nAb) in complex with the FP (purple) (PDB ID: 6CDO) (Xu et al., 2018).

(H) Crystal structure of vFP20.01 Fab (gray, a FP-elicited mouse nAb) in complex with FP (orange) (PDB ID: 6CDP) (Xu et al., 2018).

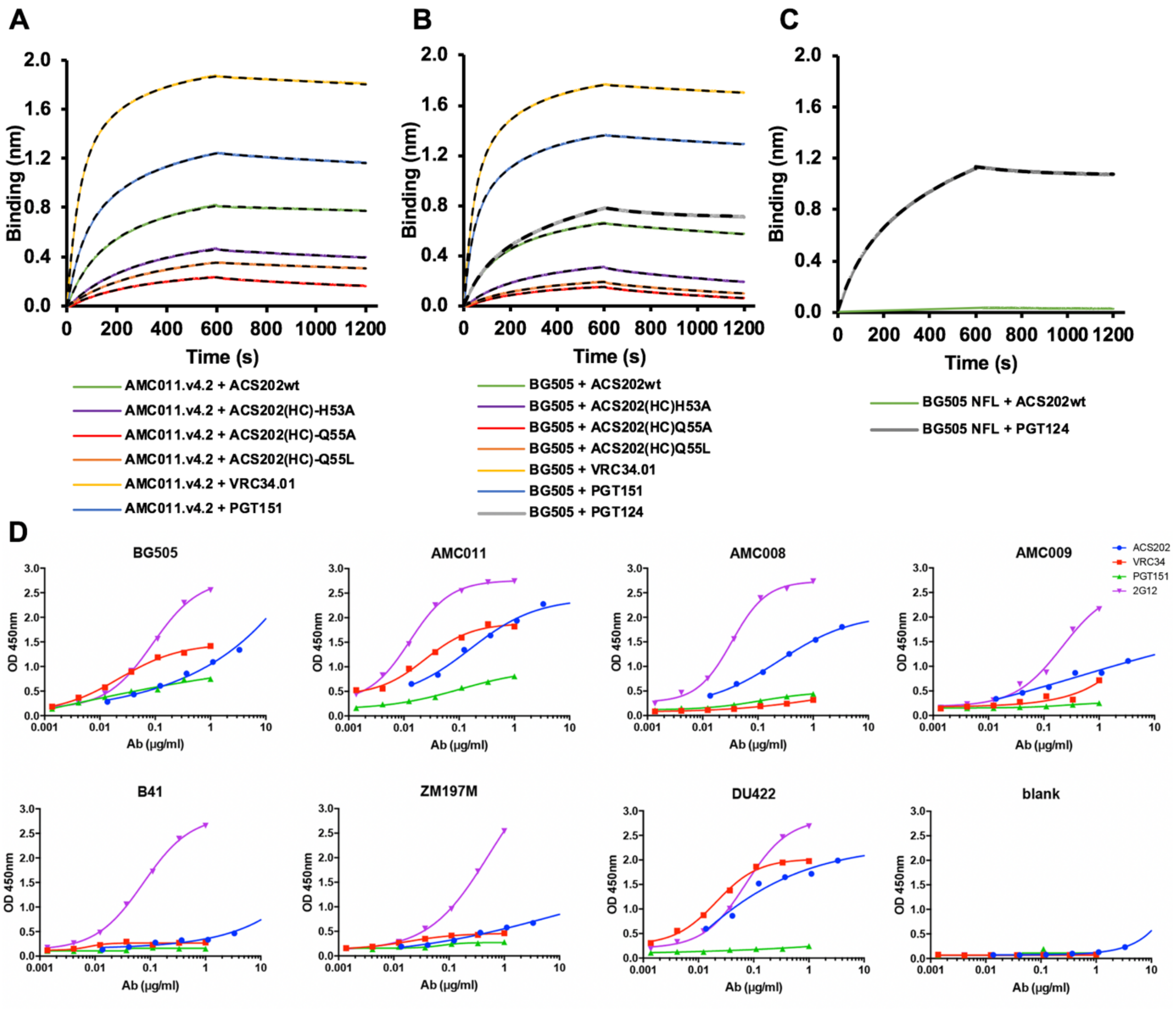

E

	85	87	88	89	90	229	241	242	243	512	513	514	514b	515	516	517	518	519
BG505	H	E	N	V	T	K	S	V	S	A	V	G	-	I	G	A	V	F
AMC011	V	E	N	V	T	N	N	V	S	A	V	G	-	I	G	A	V	F
AMC008	I	E	N	V	T	N	N	V	S	A	V	G	T	I	G	A	M	F
AMC009	V	E	N	V	T	N	N	V	S	A	V	G	A	I	G	A	V	F
B41	V	G	N	V	T	N	N	V	S	A	V	G	-	L	G	A	F	I
ZM197M	P	G	N	V	T	N	N	V	S	A	A	G	-	I	G	A	V	I
DU422	V	E	N	V	T	N	N	V	S	A	V	G	-	L	G	A	V	L

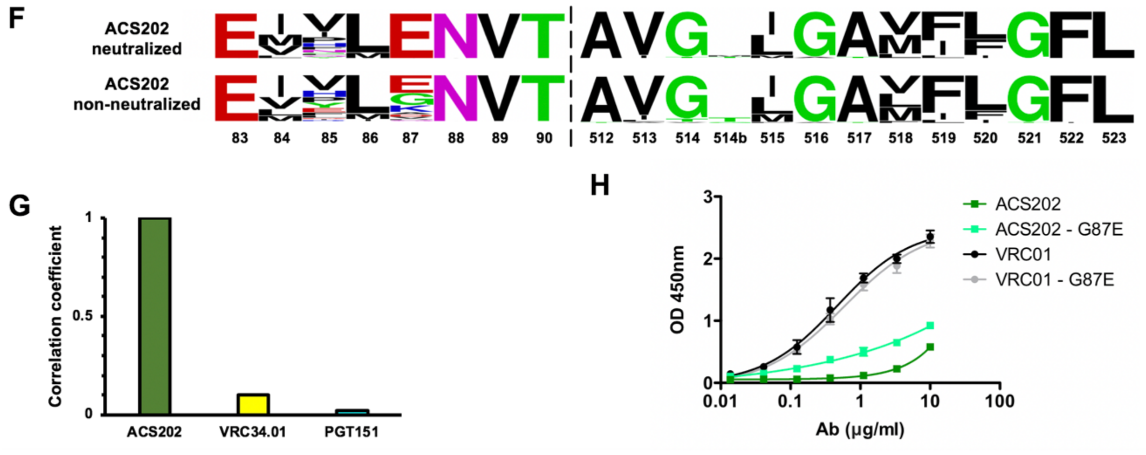

Figure S4. Binding of anti-FP antibodies to HIV-1 Env trimers. Related to Figure 2.

(A-C) Bio-layer interferometry (BLI) binding assay for anti-FP antibodies with HIV-1 Env trimers. Y-axis represents the response. Solid lines represent the response curves and black dashed lines represent the best fit models (1:2 binding model, see STAR Methods). The response curves of BG505-NFL to ACS202/PGT151/VRC34.01 were not fit due to the low response signals.

(A) Binding of VRC34.01 (yellow), PGT151 (blue), ACS202 (green) and mutants on the heavy chain including H53A (purple), Q55A (red), and Q55L (orange) to cleaved Env AMC011 SOSIP.v4.2.

(B) Binding of VRC34.01 (yellow), PGT151 (blue), PGT124 (gray), ACS202 (green) and mutants on the heavy chain including H53A (purple), Q55A (red), and Q55L (orange) to cleaved HIV-1 Env BG505 SOSIP.664.

(C) Binding of VRC34.01 (yellow), PGT151 (blue), and ACS202 (green) against non-cleaved HIV-1 Env BG505 SOSIP.664 NFL. PGT124 (gray) was used as a control.

(D) Binding of ACS202, VRC34.01, and PGT151 to different Env trimers were measured by ELISA. 2G12 was used as a control.

(E) Sequence alignment of key residues in Env proteins involved in anti-FP bnAbs recognition.

(F) Sequence logos of the ACS202-epitope region (residues 83-90 and 512-523) clustered to ACS202-neutralized and non-neutralized HIV-1 strains. A 75-virus panel was used for the neutralization assay (van Gils et al., 2016).

(G) Logistic regression analysis shows the correlation with the presence of glutamate at residue 87 and the neutralization effects of each anti-FP bnAb.

(H) Binding of bnAbs to HIV-1 Env B41 and B41-G87E was measured by ELISA. Data represent the mean ± standard error of two experiments.

Table S1. X-ray data collection and refinement statistics. Related to Figure 1.

**Data collection**	
Beamline	APS 23ID-B
Wavelength (Å)	1.0332
Space group	$P6_5$
Cell dimensions a, b, c (Å)	78.4, 78.4, 340.8
Resolution (Å)	58.31 - 2.76 (2.81 - 2.76) [a]
Total reflections	358,464 (18,377) [a]
Unique reflections	30,474 (1,555) [a]
Multiplicity	11.8 (11.8) [a]
Completeness (%)	100 (100) [a]
$<I/\sigma_I>$	5.4 (1.2) [a]
R_{merge} [b]	0.28 (1.54) [a]
R_{pim} [b]	0.09 (0.47) [a]
$CC_{1/2}$ [c]	0.99 (0.84) [a]
**Refinement statistics**	
Resolution (Å)	58.31 - 2.76
Reflections (work)	29,909
Reflections (test)	1,476
R_{cryst}/R_{free} [d]	22.2 / 25.6
Number of non-hydrogen atoms	6917
macromolecules	6727
peptides	110
ligands	20
solvent	60
Average *B*-value (Å^2)	
macromolecules	64
peptides	77
ligands	88
solvent	46
Wilson *B*-value (Å^2)	59
RMS bonds (Å)	0.006
RMS angles (°)	0.95
Ramachandran favored (%)	97.6
Ramachandran outliers (%)	0.1
PDB code	6NCP

[a] Values in parentheses are for the highest resolution shell.
[b] $R_{merge}=\Sigma_{hkl}|I-<I>|/\Sigma_{hkl}I$ and $R_{pim} = \Sigma_{hkl}\,(1/(n-1))^{1/2}\,\Sigma_i\,|\,I_{hkl,i} - <I_{hkl,i}>\,|\,/\,\Sigma_{hkl}\,\Sigma i\,I_{hkl,i}$, where $I_{hkl,i}$ is the scaled intensity of the ith measurement of reflection h, k, l, $<I_{hkl,i}>$ is the average intensity for that reflection, and *n* is the redundancy.

[c] $CC_{1/2}$ = Pearson correlation coefficient between two random half datasets.
[d] $R_{work}=\Sigma|F_{obs}-F_{calc}|/\Sigma|F_{obs}|$, where F_{obs} and F_{calc} are the observed and the calculated structure factors, respectively. R_{free} is calculated using 5% of total reflections randomly chosen and excluded from the refinement.

Table S2. Comparison of bnAbs that target the HIV-1 fusion peptide. Related to Figures 3 and 4.

	ACS202	PGT151	VRC34.01	vFP16.02	vFP20.01
Source	Human patient	Human patient	Human patient	Immunized mouse	Immunized mouse
Neutralization breadth	45% [a]	66% [b]	51% [c]	31% [c]	27% [c]
Neutralization median IC_{50} ($\mu g\ ml^{-1}$)	0.142 [a]	0.008 [b]	0.155 [c]	10.9 [c]	11.2 [c]
β-sheet with FP	Yes	Yes	No	No	No
YYYY motif	Yes	Yes	No	No	No
Antibody: protomer ratio	3:1	2:1	3:1	3:1	3:1
Conformation of FP	Extended downward	Extended upward	Extended downward	U-shape	U-shape
HC V gene	Human *HV3-30*03*	Human *HV3-30*03*	Human *HV1-2*02*	Mouse *HV1-15*01*	Mouse *HV1-15*01*
LC V gene	Human *κV1-33*01*	Human *κV2D-29*02*	Human *κV1-9*01*	Mouse *κV1-117*01*	Mouse *κV1-117*01*

[a] Neutralization assay against a panel of 75 viruses (van Gils et al., 2016).
[b] Neutralization assay against a panel of 117 viruses (Falkowska et al., 2014).
[c] Neutralization assay against a panel of 208 viruses (Xu et al., 2018).

Table S3. CryoEM data collection and model building statistics. Related to Figures 2 and 3.

Complex	AMC011 v4.2 SOSIP + ACS202 Fab	AMC011 v4.2 SOSIP + VRC34.01 Fab
EMDB accession code	EMD-0433	EMD-0434
PDB accession code	6NC2	6NC3
**Data collection**		
Microscope	FEI Titan Krios	FEI Titan Krios
Voltage (kV)	300	300
Detector	Gatan K2 Summit	Gatan K2 Summit
Recording mode	Counting	Counting
Magnification (incl. post-magnification)	48,543	48,543
Movie micrograph pixelsize (Å)	1.03	1.03
Dose rate (e^-/[(camera pixel)*s])	9.75	5.00
Number of frames per movie micrograph	50	48
Frame exposure time (ms)	200	250
Movie micrograph exposure time (s)	10	12
Total dose (e^-/Å^2)	92	56
Defocus range (μm)	1.3-2.5	0.7-2.5
**EM data processing**		
Number of movie micrographs	1,641	2,834
Number of molecular projection images in map	49,878	35,611
Symmetry	D3	D3
Map resolution (FSC 0.143; Å)	5.2	4.5
Local resolution range (Å)[1]	4.9-8.6	4.2-8.3
Map sharpening B-factor (Å^2)	-365	-125
**Structure building and validation**		

Number of atoms in deposited model		
gp120	20,688	22,014
gp41	6,264	7,272
Fab Fv	11,190	10,404
glycans	1,752	2,256
MolProbity score	1.03	1.02
Clashscore	0.7	0.9
Map correlation coefficient	0.74	0.77
EMRinger score	1.04	1.00
RMSD from ideal		
Bond length (Å)	0.01	0.02
Bond angles (˚)	1.23	1.86
Ramachandran plot		
Favored (%)	95.56	96.36
Allowed (%)	4.19	3.25
Outliers (%)	0.25	0.39
Side chain rotamer outliers (%)	0.0	0.15

[1]Relion 3.0 local resolution estimation
